# Role of Gag and lipids during HIV-1 assembly in CD4^+^ T cells and macrophages

**DOI:** 10.3389/fmicb.2014.00312

**Published:** 2014-06-25

**Authors:** Charlotte Mariani, Marion Desdouits, Cyril Favard, Philippe Benaroch, Delphine M. Muriaux

**Affiliations:** ^1^Membrane Domains and Viral Assembly, CNRS UMR-5236, Centre d'étude d'agents Pathogènes et Biotechnologies pour la SantéMontpellier, Cedex, France; ^2^Intracellular Transport and Immunity, Immunité et Cancer, Institut Curie - Inserm U932Paris, France

**Keywords:** HIV-1, lipids, assembly, Gag, CD4^+^ T cells, macrophages

## Abstract

HIV-1 is an RNA enveloped virus that preferentially infects CD4^+^ T lymphocytes and also macrophages. In CD4^+^ T cells, HIV-1 mainly buds from the host cell plasma membrane. The viral Gag polyprotein targets the plasma membrane and is the orchestrator of the HIV assembly as its expression is sufficient to promote the formation of virus-like particles carrying a lipidic envelope derived from the host cell membrane. Certain lipids are enriched in the viral membrane and are thought to play a key role in the assembly process and the envelop composition. A large body of work performed on infected CD4^+^ T cells has provided important knowledge about the assembly process and the membrane virus lipid composition. While HIV assembly and budding in macrophages is thought to follow the same general Gag-driven mechanism as in T-lymphocytes, the HIV cycle in macrophage exhibits specific features. In these cells, new virions bud from the limiting membrane of seemingly intracellular compartments, where they accumulate while remaining infectious. These structures are now often referred to as Virus Containing Compartments (VCCs). Recent studies suggest that VCCs represent intracellularly sequestered regions of the plasma membrane, but their precise nature remains elusive. The proteomic and lipidomic characterization of virions produced by T cells or macrophages has highlighted the similarity between their composition and that of the plasma membrane of producer cells, as well as their enrichment in acidic lipids, some components of raft lipids and in tetraspanin-enriched microdomains. It is likely that Gag promotes the coalescence of these components into an assembly platform from which viral budding takes place. How Gag exactly interacts with membrane lipids and what are the mechanisms involved in the interaction between the different membrane nanodomains within the assembly platform remains unclear. Here we review recent literature regarding the role of Gag and lipids on HIV-1 assembly in CD4^+^ T cells and macrophages.

## The role of gag in HIV assembly

**Figure 1:**
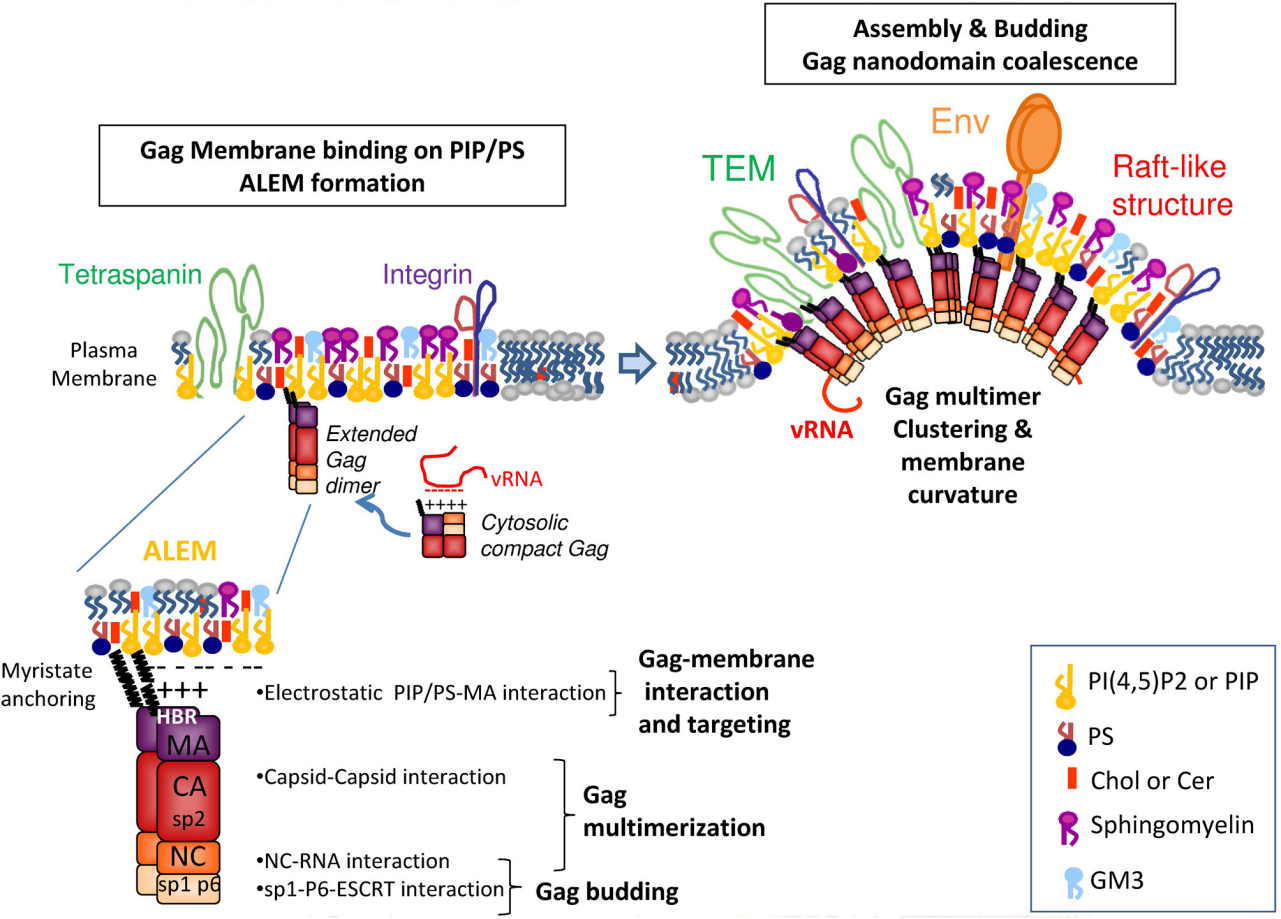
**HIV Gag assembly at the molecular level**. The viral HIV-1 Gag protein is composed of several domains: the matrix MA is myristyolated in its N-term and contains a highly basic region (HBR) that binds the lipidic membrane or the viral genomic RNA, the capsid CA that favors Gag–Gag multimerization, the NC domain that selects the viral genome and favors Gag multimerization, its C-term p6 domain that binds ESCRT proteins requires for retroviral budding and particle release and 2 spacer peptides, sp2 and sp1 required for HIV assembly and morphogenesis. In the cytosol, Gag is a dimer and adopts a compact shape in which the viral genomic RNA is in interaction with the NC and MA domains. At the cell membrane, the MA domain of Gag targets, via its HBR, the inositol head of the PI(4,5)P_2_ located at the inner leaflet of the PM and its myristate anchors the lipid bilayer. The MA domain of Gag can recruit several PI(4,5)P_2_, and PS, thanks to its basic residues. This should allow the recruitment of other Gag molecules due to Gag electrostatic membrane interaction concomitant with Gag multimerisation, via CA, and NC on the viral RNA. This process should trigger the formation of ALEM (acidic lipid enriched microdomains) in the cell membrane. These ALEM containing Gag could now join together to create higher ordered microdomains of Gag and/or microdomains containing other proteins such as Env, or tetraspanins, integrins or other cell factors found in the viral envelope. The coalescence of these microdomains containing several Gag multimers creates the HIV assembly platform.

For proper assembly of newly synthesized virions, the different viral and cellular components of HIV have to be addressed to the assembly site. The polyprotein Gag is the major structural orchestrator of the assembly process (Cimarelli and Darlix, 2002). Beside Gag itself, GagPol polyproteins, the envelop (Env) glycoprotein (Checkley et al., 2011) and the viral genomic RNA (gRNA) are recruited to the assembly site (Muriaux and Darlix, 2010; O'Carroll et al., 2013). In addition, host cell factors are required for proper trafficking of the viral constituents, as well as for virus assembly and budding. The nature of the host cell factors and their incorporation into new virions can vary depending on the producer cell and thus may impact HIV-1 infectivity (reviewed in Iordanskiy et al., 2013). Nevertheless, Gag expression alone is sufficient for virus-like particle (VLP) production. Gag is synthesized in the cytosol as a 55 kDa polyprotein comprising several domains that are cleaved into independent proteins after budding: the Matrix (MA), the Capsid (CA), the Nucleocapsid (NC) and the p6 domain (Figure 1). Gag is targeted to the site of budding where it interacts with the membrane and multimerizes. Viral assembly requires Gag–Gag interactions that can occur at different levels: MA–MA interactions upon MA-membrane interactions, CA–CA interactions and NC–NC interactions via the genomic RNA (gRNA) (Figure 1). The gRNA is recruited to the nascent viral particle via a selective interaction between its Psi encapsidation sequence and the NC domain of Gag (Muriaux and Darlix, 2010; Lu et al., 2011). The formation of VLPs at the plasma membrane (PM) of infected cells requires the myristoylation of Gag (Bryant and Ratner, 1990; Resh, 2005) and the presence of a highly basic region (HBR) in the N-term of MA for Gag anchoring into the cell membrane lipid bilayer (Chukkapalli and Ono, 2011). Other amino acids in the vicinity of MA/membrane interface could also be involved but have not yet been described. In this review, we will focus on the Gag-driven viral assembly process in HIV-1-infected T cells and macrophages. The recognition between Gag preassembling complexes and the Env will not be developed here and has been reviewed by others (Checkley et al., 2011).

## HIV producing cells: CD4^+^ T-cells and macrophages

HIV-1 mainly infects activated CD4^+^ helper T cells *in vivo* (Freed and Martin, 2001). Most infected activated T cells produce large amounts of new virions and die of apoptosis. A minority acquire a memory phenotype and progress to latently infected cells, able to survive for decades in the absence of virus production (Kuritzkes D.R., 2007). Upon arrest of therapy, these cells can be activated and participate in the rebound in HIV-1 titers, and are therefore important reservoirs of the virus. HIV-1 assembly, budding and release from CD4^+^ T cells occurs mainly at the plasma membrane (PM). In polarized CD4^+^ T-cells, HIV components for assembly, such as Gag and the genomic RNA are localized at the uropod membrane as shown by live cell fluorescence microscopy (Hatch et al., 2013; Llewellyn et al., 2013). An exception among T cells are the chronically-infected MOLT lymphoblasts in which infectious HIV-1 are found in intracellular compartments (Grigorov et al., 2006).

**Figure 2:**
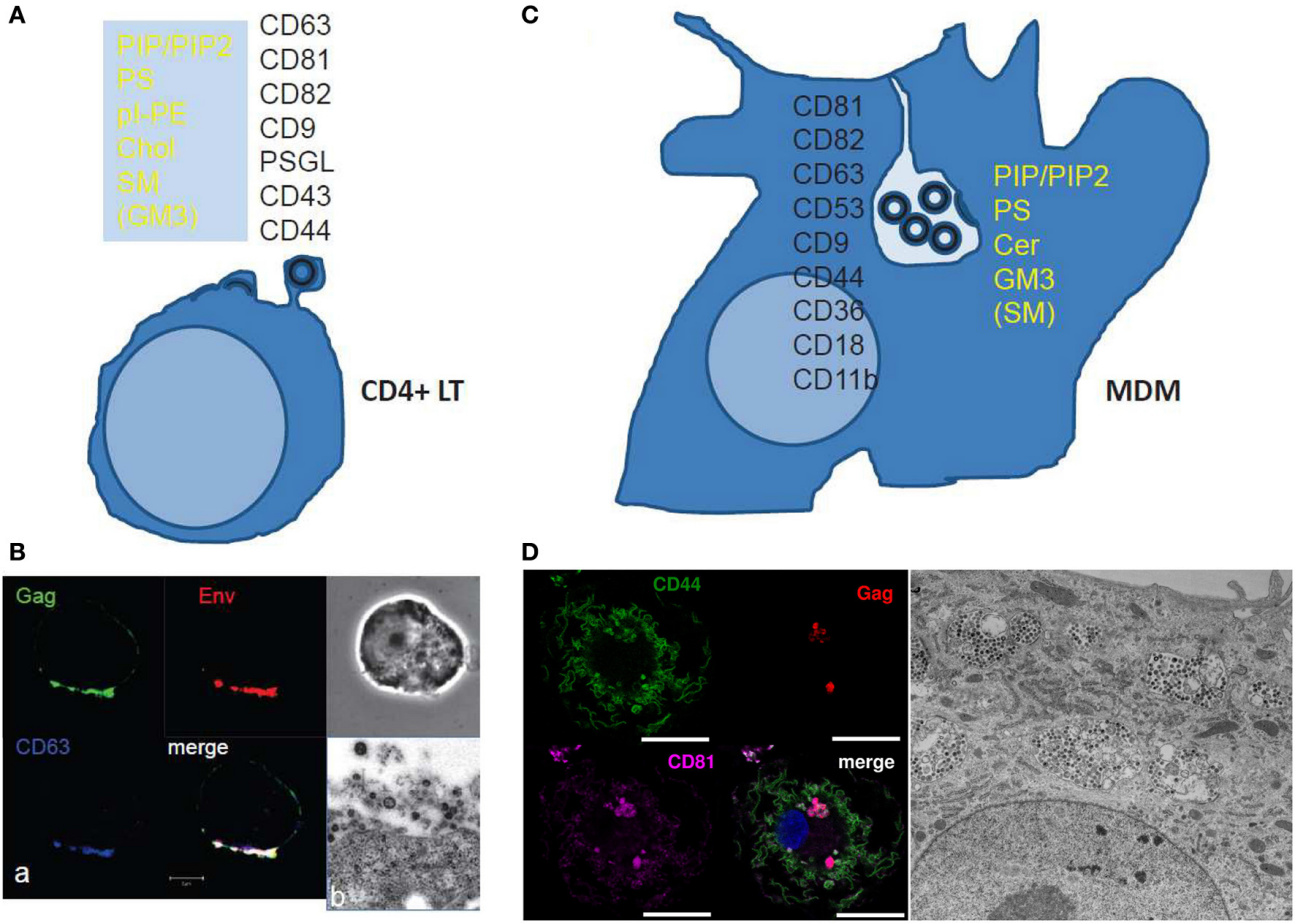
**Scheme and images of HIV assembly at the cellular level**. **(A)** Membrane cell proteins and lipid composition that are found in the virus membrane issued from CD4^+^ T cell lines or at polarized T cell uropods. From Brügger et al. (2006); Chan et al. (2008); and Lorizate et al. (2013) for the virus lipid membrane composition. From Grigorov et al. (2006, 2009); and Nydegger et al. (2006) for the virus membrane protein composition. From Llewellyn et al. (2013) for the Gag containing T cell uropod microdomains. **(B)** HIV-1 assembly in chronically infected MOLT cells as shown by immunofluorescence confocal for Gag, Env and the cell CD63 tetraspanin (a) and electron(b) microscopy. (a) HIV-1 infected MOLT cells were fixed in 3% PFA and stained for Gag with an anti-MAp17, anti-gp120, and anti-CD63 for immunofluorescence confocal imaging. (From D. Muriaux lab.) (b) HIV-1 infected MOLT cells were fixed with 2.5% glutaraldéhyde, embedded and thin-sectioned for electron microscopy imaging, as in Grigorov et al. (2009) (From P. Roingeard lab.). **(C)** The scheme shows the cell membrane proteins and lipids found associated with the viral membrane of HIV-1 issued from monocyte derived macrophages. From Chertova et al. (2006); Chan et al. (2008); Lorizate et al. (2013). **(D)** HIV assembly in VCC in MDM as shown by immunofluorescence confocal microscopy and electron microscopy. MDM 4 days post-infection with WT HIV-1 NL-AD8, stained by immunofluorescence for CD44 (green), Gag (red), and CD81 (magenta). The nucleus is stained with DAPI. Bar: 20 μm. CD44 and CD81 are present at the plasma membrane but also in intracellular compartments were they co-localize with Gag in infected MDM. (From P.Benaroch lab). For electron microscopy, kindly provided by Mabel Jouve, MDM 15 days post-infection with WT HIV-1 NL-AD8 were fixed, embedded in epon, and ultrathin sections were contrasted with uranyl acetate and lead citrate. Bar: 2 μm.

Macrophages are the other main cellular target of HIV. They play important roles during HIV infection and AIDS progression due to their specific features (Koppensteiner et al., 2012). Infected macrophages have been found in all tissues such as the vaginal mucosa, the brain and the lung (Gartner et al., 1986; Shen et al., 2009; Jambo et al., 2014), where they remain infectious for long periods of time (Sharova et al., 2005). In contrast with T cells, macrophages resist HIV-induced cytopathic effects for months *in vitro* as well as *in vivo* (Koppensteiner et al., 2012). They accumulate virions in large intracytoplasmic vacuoles (Orenstein et al., 1988), that are often referred to as VCCs (Virus-Containing Compartments) (Figure 2C) or IPMCS (Intracellular Plasma Membrane-Connected Compartments) (Mlcochova et al., 2013). Electron microscopy studies of infected macrophages revealed budding profiles at the limiting membrane of the VCC and the presence of immature virion in the lumen of the compartments, demonstrating that VCCs represent the site of HIV assembly in macrophages (Orenstein, 1998; Raposo et al., 2002; Pelchen-Matthews, 2003; Jouve et al., 2007). Newly formed viral particles accumulate in the VCC lumen and VCCs tend therefore to fill up with time (Gaudin et al., 2013). Early on, VCCs were classified as late endosomes or multivesicular bodies because of their endosomal markers and their morphology (Raposo et al., 2002; Pelchen-Matthews, 2003). However, they were later shown to be inaccessible to BSA-gold, devoid of EEA1 (Deneka et al., 2007; Jouve et al., 2007) and to possess a neutral luminal pH (Jouve et al., 2007), suggesting that they were not true endosomes. The lumen of the VCCs can be transiently accessible to extracellular molecules (Deneka et al., 2007; Berre et al., 2013; Gaudin et al., 2013) possibly through direct tubular connections to the PM (Welsch et al., 2007; Bennett et al., 2009). VCCs are now regarded as specialized, intracellular sequestered portions of the PM, possibly with dynamic connections to the extracellular milieu (Gaudin et al., 2013; Mlcochova et al., 2013), for reviews see Benaroch et al. (2010) and Tan and Sattentau (2013).

The two main cellular targets of HIV, CD4^+^ T cells and macrophages, exhibit very different morphology, function and physiology. The assembly site of HIV in both cell types appears different : the PM of small round T-lymphocytes versus the internal convoluted membrane meshwork of large macrophages (Figure 2). This raises the question of the cell specificity for the mechanism(s) underlying virus assembly and for the lipid membrane composition of produced virions. Most studies pertaining to the role of Gag and lipids in HIV assembly in live cells used T-cell lines or adherent epithelial cell lines, with only few results confirmed in human primary T-cells or monocyte-derived macrophages (MDM), the classical culture model for macrophages. However, we will see below that most observations and mechanistic hypotheses regarding the role of membrane lipids in HIV assembly can apply for T-cells and macrophages.

## Composition of HIV assembly sites in T cells and macrophages

### Tetraspanin-enriched microdomains

In primary T-cells and T-cell lines, Gag accumulates in specific domains of the PM containing the tetraspanins CD9, CD63, CD81, and CD82 that are referred to as Tetraspanin Enriched Microdomains (TEM) (Booth et al., 2006; Grigorov et al., 2006; Nydegger et al., 2006). These tetraspanins are present in subdomains of the PM of HIV-infected T cells together with Gag and Env as shown by immunofluorescence analysis and immunoprecipitation (Jolly and Sattentau, 2007; Grigorov et al., 2009) (Figures 2A,B). In addition, virions released and purified from these cells are associated with the same tetraspanins (Grigorov et al., 2009). HIV-infected and polarized T-cells possess “specific uropod-directed microdomains” that contain PSGL-1 (P-Selectin Glyco- protein Ligand 1), CD43 and CD44 (Figure 2A) where Gag localizes due to its NC and MA regions (Llewellyn et al., 2013).

In infected macrophages, TEMs are also a component of the assembly site of HIV and the same tetraspanins are enriched at the VCC limiting membrane as revealed by immunofluorescence and electron microscopy (Figure 2C) (Deneka et al., 2007; Gaudin et al., 2013; Mlcochova et al., 2013). This is also reflected by the composition of virions produced by macrophages, which harbor several tetraspanins (CD9, CD81, CD82, CD53, CD63) as assessed by mass spectrometry (Chertova et al., 2006). These virions also contain other proteins enriched in the VCCs, such as CD44, CD18, and CD11b, further highlighting that the envelope of the virus derives from the membrane of the VCC (Figures 2C,D). Interestingly, the limiting membrane of the VCC as well as the virions present in the VCC of infected macrophages contains the scavenger receptor CD36, which represents so far the first macrophage specific marker of the viral assembly site as compared to T lymphocytes (Berre et al., 2013).

### Virus membrane lipid composition

Lipidomic analysis of HIV virions has provided important information regarding the nature of the membrane domains where the viral budding takes place (Aloia et al., 1993; Brügger et al., 2006; Chan et al., 2008; Lorizate et al., 2013). Early work revealed that HIV-1 and HIV-2 virions were enriched in cholesterol when compared to the PM of producer cells H9 T-cells, suggesting that HIV buds from specific domains of the PM (Aloia et al., 1993). Later, Chan et al. showed that virions from T cells were mainly enriched in acidic lipids (PI, PIP_2_, PS), GM3, cholesterol and SM (Chan et al., 2008), a composition close to that of “rafts-like” domains. However, the HIV assembly site exhibits some differences with these domains since the GM1 raft lipid and the CD55 raft protein were only transiently found trapped in viral assembly sites, at least in adherent cell lines (Krementsov et al., 2010). The dynamics of other lipids have not been studied during Gag assembly in living primary host cells and should be investigated in order to validate the role of lipids in HIV assembly and budding or the role of Gag in the formation of lipidic domains. A recent study done on MT4 T cells showed an enrichment in PS, PI, pl-PE, PG, and hexCer but not Cholesterol in the virus particles when compared to the PM (Lorizate et al., 2013). This suggested that the HIV envelope composition can vary as a function of the membrane composition of the producer cell or depends on experimental conditions, such as techniques used to purify the PM (Lorizate et al., 2013) or to separate viral particles from microvesicles shed by the cells (Chertova et al., 2006; Chan et al., 2008; Coren et al., 2008). However, a constant observation is the enrichment in glycosphingolipids (Cer or SM) and/or Cholesterol that could locally order the virion bud membrane and in an acidic phospholipid (PI, PIP_2_, or PS) that is required for Gag membrane recruitment (see below). Figure 2A summarizes the lipidic and proteic enrichment of HIV envelope composition in T-lymphocytes and MDM.

Only one study so far investigated the lipidomic composition of HIV virions produced by macrophages (Chan et al., 2008). It showed that, when compared to the PM of the macrophages, viral particles are enriched in phosphorylated derivatives of phosphatidylinositol (PIP, PIP_2_), the glycosphingolipid GM3 and to a lesser extend sphingomyelin but not in cholesterol. HIV virions from macrophages are also enriched in ceramide, which may be a sign of coalescence of other lipid nanodomains into bigger domains at the level of the assembly platform. Importantly, Chan et al. compared HIV virions produced by T-cells and macrophages. Both types of virions exhibited a very similar lipidic composition while the global PM composition was different in macrophages as compared to T-cells (Chan et al., 2008). These data suggest that HIV is able to reach or to create a favorable lipidic environment containing the components necessary for its assembly and budding in both cell types.

Thus, in macrophages as in T cells, HIV assembles at and buds from specific membranes enriched in acidic lipids, sterols and/or glycosphingolipids (reviewed in Kerviel et al., 2013). Nevertheless, the fine mechanism underlying Gag targeting to such PM domains remains unclear.

## Gag targeting to the viral budding site

Gag synthesis occurs in the cytoplasm where it is myristoylated (Bryant and Ratner, 1990). Gag selectively interacts with the genomic RNA from which it has been translated (de Breyne et al., 2013). Then Gag is targeted to the cell PM (Kutluay and Bieniasz, 2010) either by transport as minimal Gag-RNA complexes or simply by diffusion. At this point, cytosolic Gag is probably in a low dimerization state (Kutluay and Bieniasz, 2010) and in a compact conformation likely in interaction with the viral RNA (Figure 1) (Datta et al., 2007; Munro et al., 2014) and should reach the plasma membrane for assembly.

### Gag interaction with the plasma membrane: a role for PI(4,5)P_2_

The MA domain of Gag can bind lipids at the inner surface of the cell PM where Gag multimerizes, thanks to its myristate (Bryant and Ratner, 1990) and its Highly Basic Region (HBR) (Ono, 2009). Based on biophysical experiments, conformations of a monomeric form of HIV-1 Gag were analyzed in solution, and the results revealed that Gag can adopt a compact conformation (Datta et al., 2007). Single molecule FRET experiments and FCS revealed that upon assembly into VLP, Gag undergoes a conformational transition from compact to an extended form (Munro et al., 2014). It has been proposed that upon its interaction with membrane phospholipids and during its multimerization, Gag adopts an extended conformation, similar to the one observed in immature virus. This process is triggered by the interaction of the HBR at the N-ter of MA with the PM PI(4,5)P_2_ phospholipid (Ono et al., 2004a; Saad et al., 2006; Chukkapalli et al., 2008). In addition, the genomic RNA and the PI(4,5)P_2_ are probably in competition for the binding of the MA HBR (Chukkapalli et al., 2010). This suggests that under its compact conformation, Gag is able to interact with the genomic RNA through the MA HBR as well as through the NC and as soon as Gag is in the vicinity of a PI(4,5)P_2_ containing membrane, it switches to the extended conformation which the HBR interacts with PI(4,5)P_2_. Thus, it appears that MA-membrane binding requires the anchoring of the N-term MA myristate in the membrane lipid bilayer, a change of Gag conformation from a compact to an extended rod-like shape, and the electrostatic interaction between the charged sugar head of phospholipids (such as PI(4,5)P_2_ and PS) and the MA HBR (Figure 1).

PI(4,5)P_2_ facilitates Gag-membrane binding to the plasma membrane in T cells and is required for efficient virus release (Monde et al., 2011). However, in T cells engineered to express low levels of PI(4,5)P_2_, the virus particle production can still occur, suggesting that the MA domain of Gag can probably interact with other charged acidic lipids, such as PS, as reported by lipidomic analysis of HIV virions (Lorizate et al., 2013). Interestingly, in these T cells, the virus adapted to compensate the lack of PI(4,5)P_2_ by a “charged” mutation in MA (L74R) that enhances virus infectivity (Monde et al., 2011). The role for PI(4,5)P_2_ dependence for virus release remains unclear and may involve other cellular lipids or proteins or a cell signaling cascade. This interaction regulates the proper targeting of the viral components to the assembly site. Indeed, mutations of the HBR MA domain induce re-localization of Gag from the PM to CD63+ intracellular compartments in HeLa and in T cells (Ono et al., 2004b).

In macrophages, both WT and MA-mutant Gag co-localize with a subpopulation of CD63^+^ compartments (Ono et al., 2004b; Gousset et al., 2008). However, it is unclear whether these CD63^+^ compartments represented true VCCs, since few other VCC specific markers were used in these studies (Ono et al., 2004a; Gousset et al., 2008). Importantly, PI(4,5)P_2_ was detected at the limiting membrane of intracellular CD81+, CD44+, or Gag+ compartments and at the PM in infected macrophages (Mlcochova et al., 2013). Together, these results suggest that the basic region of the MA domain may contribute to Gag targeting to the VCC through phospholipid binding, in MDM as in T-cells. In addition, other unknown factors may be required to regulate this targeting (Ono et al., 2004b; Gousset et al., 2008; Mlcochova et al., 2013).

### Other retroviruses

The mechanism by which the HBR of retroviral MA (Murray et al., 2005) binds the PM PI(4,5)P_2_ phospholipid is likely to be general for many retroviruses such as HIV-1 (Ono et al., 2004a; Saad et al., 2006; Chukkapalli et al., 2008), HIV-2 (Saad et al., 2008), EIAV (Chen et al., 2008), MLV (Hamard-Peron et al., 2010), and MPMV (Prchal et al., 2012). It is controversial for Rous Sarcoma Virus (RSV) as some authors have found that *in vitro* RSV MA binding to membranes requires acidic lipids like PS but not PI(4,5)P_2_ (Chan et al., 2011). In addition, no effect of the phosphatase-mediated depletion of PI(4,5)P_2_ was reported on RSV Gag cellular localization and VLP production. In contrary, another study described, in a cellular context, that the presence of PI(4,5)P_2_, and/or PI(3,4,5)P_3_, is required for RSV Gag targeting to the PM and for virus release in a NC-dependent Gag multimerization manner (Nadaraia-Hoke et al., 2013). In the case of EIAV, MA has greater affinity for PI(3)P than PI(4,5)P_2_, as shown *in vitro* by NMR lipid titration. Interfering with the metabolism of PI(3)P, but not of PI(4,5)P_2_, prevents EIAV assembly and release, indicating a slight difference of PI recognition for this retrovirus compared to others (Fernandes et al., 2011). Only one retroviral Gag seems to make an exception, as HTLV-1 MA does not require PI(4,5)P_2_ interaction to trigger Gag targeting and membrane binding (Inlora et al., 2011).

## Gag-driven construction of the assembly platform

Differences in lipid composition between HIV envelop and the host cell PM suggest, as mentioned earlier, that the virus buds from a specific Gag-containing membrane domain. Gag could either bind these pre-formed lipidic and proteic microdomains at the PM, or induce their formation by segregating the components through electrostatic interactions and Gag multimerization.

### Acidic lipid enriched microdomains (ALEM)

We hypothesize that Gag alone or minimal Gag-RNA complexes are targeted to the PM not only by the electrostatic interaction of the HBR region of MA with the PI(4,5)P_2_/PS lipids, but also by Gag multimerization via its CA/CA interaction and NC/RNA interaction. The stabilization of the MA—membrane interaction appears to rely on the insertion of the myristate into the lipid bilayer (Charlier et al., 2014), as well on the interaction of the HBR motif with the sugar head of PI(4,5)P_2_ and consequently on Gag multimerization. Other cellular cofactors of membrane microdomain formation such as cortical actin (unpublished data) or tetraspanin web (Thali, 2009) could contribute to the stabilization of Gag multimers at the cell PM. We propose that upon Gag—membrane interaction, Gag multimerization triggers the formation of acidic lipid enriched microdomains (ALEM) at the inner leaflet of the cell membrane (Kerviel et al., 2013) as it has been shown by dynamic coarse grained modeling of HIV-1 MA anchoring in a lipidic membrane (Charlier et al., 2014). The generation of ALEM by Gag itself is likely due to lipid sequestering induced by electrostatic interaction as reported before for other cellular proteins (van den Bogaart et al., 2011). This early event certainly generates nanodomains smaller than the size of a virus bud and different from rafts. Then viral assembly could propagate either by expansion of these nanodomains or by coalescence of several Gag-enriched membrane nanodomains to form the final viral bud.

### Microdomains coalescence

The formation of ALEM by higher order Gag multimer formation could then drain other microdomains of the external leaflet of the cell PM such as tetraspanin-enriched microdomains (TEM) or rafts, that contain other lipids like Ceramide, Cholesterol, Sphingomyelin and proteins such as tetraspanins (Yanez-Mo et al., 2009) or the embedded Env glycoproteins (Checkley et al., 2011). Coalescence of nanodomains has been reported for Gag with “rafts-like” membrane domains and TEM in Hela cells (Krementsov et al., 2010; Hogue et al., 2011). This model is supported by the work of Ono and co-workers, with antibody-mediated copatching and FRET assays (Hogue et al., 2011) conducted on adherent Hela cells expressing HIV-1. Interactions between Gag and the inner leaflet of PM appear to induce the coalescence of TEM and lipid raft at the viral assembly site. This is also in line with a model where Gag does not associate with pre-existing virus-sized microdomains organized by cellular factors, but rather functions as a microdomain-organizing factor to create novel virus-induced microdomains in a stepwise manner during the course of assembly (Krementsov et al., 2010; Hogue et al., 2011; Kerviel et al., 2013).

In this model (Figure 1), Gag recruits PI(4,5)P_2_ upon its binding to the membrane, then its multimerization induces the formation of ALEM in the inner leaflet of the membrane, which further triggers the coalescence of other nanodomains of the outer leaflet of the membrane, such as lipid rafts and TEM (Kerviel et al., 2013; Charlier et al., 2014).

In polarized CD4^+^ T cells, such advanced studies have not been done, but Ono and co-workers have shown that HIV-1 Gag assembly occurs in uropod-specific microdomains and that this Gag localization depends on higher order NC dependent multimerization of Gag (Llewellyn et al., 2010), in agreement with the model proposed above. In macrophages, studies that pertain to the molecular interactions driving the construction of the assembly platform are still lacking. Considering that Gag assembly is a sequential process and several molecules are required to form higher ordered microdomains, Gag-acidic lipid nanodomains certainly fuse together. In addition, they can get enriched in other lipids via the coalescence with other surrounding nanodomains containing cell membrane proteins such as CD81 tetraspanin (Nydegger et al., 2006; Grigorov et al., 2009) or other lipids from the upper bilayer, such as SM and GM3 (Chan et al., 2008).

### A role for cholesterol?

Gag could directly sense other lipids than PIP_2_, like cholesterol (Dick et al., 2012). Depletion of cellular cholesterol markedly and specifically reduced HIV-1 particle production (Ono and Freed, 2001). Drug-induced redistribution of the cholestorol from the PM to endosomes led to relocation of Gag from the PM to intracellular MVB-like CD63^+^ compartments (Lindwasser and Resh, 2004), suggesting a possible role for cholesterol in Gag targeting to assembly sites. However, Gag contains no cholesterol binding motif, unlike other viral proteins with roles in entry or morphogenesis (Schroeder, 2010). Several studies have proposed that HIV-1 regulates the levels of cellular cholesterol, presumably to achieve efficient viral egress. A Nef-induced decrease of the ABCA1-dependent efflux of cholesterol to the Apolipoprotein A1 has been reported (Mujawar et al., 2006; Morrow et al., 2010), leading to increased cholesterol in lipid rafts (Cui et al., 2012). This mechanism is likely involved in HIV-associated dyslipidemia as observed in macaques infected by SIV (Asztalos et al., 2010). However, Nef had no impact on virus content in cholesterol in MT4 T cells (Brügger et al., 2007). Thus, the role of cholesterol in virus budding and infectivity remains to be assessed.

## Modulation of calcium signaling in HIV-1 assembly and release

The PI(4,5)P_2_ represents 1–2% of the total phospholipids at the inner leaflet of the cell PM, and it is a key regulator of several cellular processes taking place at the cell PM, such as endocytosis, exocytosis, cytoskeleton attachment and activation of enzymes (reviewed in McLaughlin and Murray, 2005). PI(4,5)P_2_ anchors cellular proteins to the PM through Pleckstrin Homology domains that can activate different ion channels located at the PM (Suh and Hille, 2005). PI(4,5)P_2_ is also the precursor of second messengers such as inositol(1,4,5)triphosphate (IP_3_) or diacylglycerol (DAG). IP_3_ can regulate release of Ca^2+^ from internal storages; DAG can activate the protein kinase C (PKC). In the course of HIV assembly and budding, it was suggested a role for intracellular Ca^2+^ increase in boosting virus release (Grigorov et al., 2006; Perlman and Resh, 2006). In addition, calmodulin, CaM, a regulator of intracellular Ca^2+^ concentration, interacts with the N-terminal domain of HIV-1 MA as shown, *in vitro*, by NMR and biochemical experiments (Samal et al., 2011; Vlach et al., 2014). This interaction depends on Ca^2+^ and is able to induce the MA myristate exposure and an extended MA conformational change (Chow et al., 2010; Ghanam et al., 2010; Taylor et al., 2012). In cells, Carter and co-workers have shown that HIV-1 Gag induces Ca^2+^ release from intracellular compartments, probably from the endoplasmic reticulum, by activating the cascade PI(4,5)P_2_-PLC-IP_3_-IP_3_R (Ehrlich et al., 2010; Ehrlich and Carter, 2012). IP_3_ receptor and Sprouty2, also regulators of Ca^2+^ signaling, are required for HIV Gag particle release (Ehrlich et al., 2011). Thus, it appears that in addition to the requirement of the PI(4,5)P_2_ as a co-factor for HIV-1 Gag targeting to the PM, related lipid metabolism and second messengers or Ca^2+^ signaling are involved in virus particle release. These findings open a new door in the regulation of HIV-1 particle production.

## Conclusion

CD4^+^ T lymphocytes and macrophages are very different in size, shape and immune functions. The HIV-1 virions produced by CD4^+^ T cells or macrophages carry specific lipids and cellular proteins, incorporated during virus assembly. The cellular components identified so far appear quite similar in both cell types, suggesting that, at the molecular level, Gag-membrane interaction with lipids (and proteins) occurs in a similar way during assembly. This is in line with a model where Gag contains most of the determinants for selecting or creating the best lipidic environment for viral bud formation. We propose that Gag multimerization will generate an assembly platform containing specific lipid membrane nanodomains through its interaction with selective lipids. The creation of this assembly platform in the inner layer of the PM, probably recruits the nanodomains enriched in other proteo-lipid domains present in the upper layer.

Finally, all the components involved in the PM structure and signaling (i.e., lipid metabolism, calcium mobilization and/or actin cytoskeletal reorganization) may contribute to the assembly process of enveloped viruses. Deciphering the complex process of assembly, will require the identification of additional cellular proteins that have yet to be discovered.

### Conflict of interest statement

The authors declare that the research was conducted in the absence of any commercial or financial relationships that could be construed as a potential conflict of interest.
